# Treatment Patterns in Polyarticular Juvenile Idiopathic Arthritis: A Retrospective Observational Health Claims Data Study

**DOI:** 10.3390/life14060712

**Published:** 2024-05-31

**Authors:** Gerd Horneff, Julia Borchert, Joanna Diesing, Pascal Klaus, Ria Heinrich, Heike Dally, Christine Hagemann, Simon Kock, Tonio Schönfelder

**Affiliations:** 1 Department of General Paediatrics, Asklepios Clinic Sankt Augustin, 53757 Sankt Augustin, Germany; g.horneff@asklepios.com; 2 Department of Paediatric and Adolescents Medicine, University Hospital of Cologne, 50937 Cologne, Germany; 3 Scientific Institute for Health Economics and Health System Research (WIG2 GmbH), 04109 Leipzig, Germany; julia.borchert@wig2.de (J.B.); joanna.diesing@wig2.de (J.D.); ria.heinrich@wig2.de (R.H.); 4 Pfizer Pharma GmbH, Friedrichstraße 110, 10117 Berlin, Germany; pascal.klaus@pfizer.com (P.K.); heike.dally@pfizer.com (H.D.); christine.hagemann@gmx.de (C.H.); 5 InGef—Institut für Angewandte Gesundheitsforschung Berlin GmbH, Otto-Ostrowski-Straße 5, 10249 Berlin, Germany; s.kock@posteo.de; 6 Lehrstuhl Gesundheitswissenschaften/Public Health, Technische Universität Dresden, 01062 Dresden, Germany

**Keywords:** arthritis, disease-modifying antirheumatic drugs, juvenile arthritis, JIA, therapeutics, treatment patterns

## Abstract

(1) Background: Achieving inactive disease decreases long-term joint damage in patients with polyarticular juvenile idiopathic arthritis (polyJIA). The aim of our study was to describe average time to treatment and medication changes over time. (2) Methods: Incident polyJIA patients were retrospectively identified in the InGef and WIG2 longitudinal health claims databases. Drug escalation level changes were evaluated longitudinally and cross-sectionally across three years, as follows: no treatment, glucocorticoids (GCs) and/or non-steroidal anti-inflammatory drugs (NSAIDs), conventional synthetic disease-modifying antirheumatic drugs (csDMARDs), and biological disease-modifying antirheumatic drugs (bDMARDs). (3) Results: On average, newly diagnosed polyJIA patients received their first csDMARD prescription after 128 days and their first bDMARD prescription after 327 days. More patients were treated with csDMARDs than with bDMARDs at diagnosis; however, 24% and 12% (InGef and WIG2 databases, respectively) had no JIA treatment. After three years, 45% and 31% were not taking any treatments, while 18% and 36% were prescribed bDMARDs. Among patients initiating bDMARDs, most continued treatment for three years, with some switching to csDMARDs or discontinuing treatment. Patients treated only with csDMARDs took them longer, compared to those additionally taking other DMARDs. Patients treated with bDMARDs took them about twice as long as the csDMARDs they took prior. (4) Conclusion: A substantial number of patients with polyJIA are not treated as intensively as guidelines recommend.

## 1. Introduction

Juvenile idiopathic arthritis (JIA) therapeutic management aims to achieve and maintain rapid disease remission, or at least low disease activity, by limiting inflammation and symptoms. The ultimate goal is to improve health-related quality of life, and prevent irreversible damage [1,2]; achieving periods of inactive disease, even for short time periods, improves long-term prognosis [3].

Depending on the JIA category, current treatment options include non-steroidal anti-inflammatory drugs (NSAIDs), glucocorticoids (GCs), conventional synthetic disease-modifying antirheumatic drugs (csDMARDs), biological disease-modifying antirheumatic drugs (bDMARDs), and targeted small molecules, specifically Janus kinase inhibitors (JAKis) [2,4,5,6,7,8,9,10,11].

When JIA initially affects multiple joints, it is referred to as polyarticular JIA (polyJIA), and a polyarticular course of JIA can occur in most ILAR categories [12]. PolyJIA is difficult to treat and has a worse prognosis (compared to a non-polyarticular course of JIA), and these patients are less likely to achieve disease remission [13,14,15,16,17]. Achieving inactive disease at least once in the first five years of polyJIA is associated with less long-term joint damage; therefore, early aggressive and effective disease treatment is important in achieving this goal [3]. When remission with csDMARDs, specifically methotrexate, cannot be reached, bDMARDs are recommended [2,7,8,9]. NSAIDs and GCs can be used additionally to manage symptoms and/or in case of high disease activity [2].

Little is known about treatment patterns in patients with polyJIA. There is a lack of real-world data on treatment as per current guidelines, such as when csDMARDs or bDMARDs are initiated, and how long polyJIA patients remain on these treatments. The aim of our study was to analyze real-world healthcare claims data descriptively, to determine time to treatment from incident polyJIA diagnosis and changes in medications over time.

## 2. Materials and Methods

### 2.1. Database

This was a retrospective, observational study using two different longitudinal health claims databases: the WIG2 (Scientific Institute for Health Economics and Health System Research GmbH, Leipzig, Germany) and the InGef (Institut für angewandte Gesundheitsforschung Berlin GmbH, Berlin, Germany) research databases. A representative sample (in terms of age and gender) of the statutory health insurance (SHI) population per year in Germany from each of the WIG2 (about 3.5 million patients) [18,19] and InGef (about 4 million patients) [20,21,22,23] research databases was selected for analyses. Each database contains non-overlapping SHI patient data. Due to data protection regulations, database results had to be considered separately; data were only available to, and could be analyzed by, each institute separately. These claims data were collected for billing purposes and can be used for research purposes provided in anonymous form. According to the 10th chapter of book V of social code in Germany (SGB V, Sozialgesetzbuch), approval from an institutional review board is not required.

These databases have been used in previously peer-reviewed published research, with a detailed description of the databases and their capabilities [24].

### 2.2. Study Design and Population

**Figure 1:**
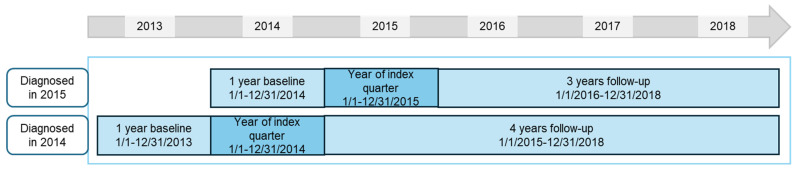
Study design for patients diagnosed in 2014 and 2015; analysis was performed separately on each of WIG2 and InGef databases.

In each database, the following patients were included: aged 2 to 15 years, diagnosed in 2014 and 2015 (1st of January to 31st of December in each year) with incident polyJIA (ICD-10 diagnosis codes M08.0, juvenile chronic polyarthritis, adult type, with or without rheumatoid factor detection, and M08.3, juvenile chronic arthritis (seronegative), polyarticular form [25]), with at least 12 quarters continuous follow-up data (or deceased). Follow-up was at least up to and including 2017 or 2018, depending on the year of index diagnosis (Figure 1). We used the year prior to diagnosis year (2013 and 2014) to rule out these diagnosis codes and ensure the patient was newly diagnosed with polyJIA and assumed to have incident disease. Our database is a claims database for billing purposes, without diagnostic results; to increase the certainty of the diagnosis, patients had to have at least one main inpatient or at least two verified outpatient diagnoses, in at least two different quarters, in 2014 and/or 2015. This case definition has been described in a previous publication of results on incidence, prevalence, and comorbidities from this study [24].

We analyzed treatment patterns in patients diagnosed in 2014 (follow-up 2015 to 2018) and in patients diagnosed in 2015 (follow-up 2016 to 2018). These populations were disjoint, with patients meeting inclusion criteria in both years having been only counted in the 2014 group. We also analyzed a pooled population per database of patients diagnosed in both 2014 and 2015, in which the fourth year of follow-up (in patients diagnosed in 2014) was not included.

#### 2.2.1. Most Frequently Prescribed and Predefined Drugs and Times to Prescription (in Days)

The top 20 most frequently prescribed drugs (identified by Anatomical Therapeutic Chemical (ATC) codes [26]) were evaluated in each of the baseline, index quarter, and follow-up periods, among all patients diagnosed in 2014 and 2015. The baseline period was the four quarters prior to the index quarter, the index quarter was that in which the first polyJIA diagnosis was documented, and follow-up was the 12 quarters for patients diagnosed in 2015, 16 quarters for patients diagnosed in 2014.

We evaluated polyJIA patients with documented prescriptions of predefined drugs of interest (NSAID, GC, csDMARD, bDMARD, JAKi), as a proportion of all polyJIA patients, in the respective timeframe. We used ATC codes (see Supplemental Tables S1–S5), evaluating the number of different types of csDMARDs and bDMARDs prescribed to patients in our databases and observing any pattern change from 2014 to 2015.

We calculated the time from polyJIA diagnosis to the first csDMARD or bDMARD/JAKi prescription as days from the start of the diagnosis quarter to the first prescription. Days from the first csDMARD prescription to the first bDMARD/JAKi prescription were also measured.

#### 2.2.2. Cross-Sectional and Longitudinal Treatment Escalations

Pooled data from patients diagnosed in both years (2014 and 2015) were used for drug treatment escalation and predefined treatment pattern analyses.

The prescription date was used to assign a prescription to a given half-year (HY) period (by quarter, either Q1 + Q2 or Q3 + Q4 of the calendar year) relative to the index quarter, because this provided the best temporal resolution, given the available sample size. For each HY period, the highest drug treatment escalation was determined based on the following hierarchy of drug treatment escalation categories, listed from highest to lowest: bDMARDs/JAKis, csDMARDs, GCs, NSAIDs.

If the patient had none of these prescriptions, they were categorized as such. These levels were assessed cross-sectionally and longitudinally. In the cross-sectional analysis, the distribution of patients to the four different treatment levels in each HY period was examined.

In the longitudinal analysis, switches and additions were considered, in addition to the distribution of treatment levels. All escalation level combinations over time were analyzed to reconstruct treatment patterns.

#### 2.2.3. Duration of Drug Exposure

Drug exposure in days was calculated for the initial treatment, starting from the index quarter and including up to three following treatments during the 12- or 16-quarter follow-up. Four different treatment patterns and the duration of drug use were defined: patients taking only NSAIDs or GCs; patients treated with only csDMARD; patients who started a bDMARD after taking a csDMARD; and finally, patients who switched from a first bDMARD to a second bDMARD, after having initially taken a csDMARD. When we refer to a csDMARD switch to a bDMARD, this is irrespective of whether the csDMARD therapy was continued or not after the bDMARD therapy was started.

## 3. Results

Applying the inclusion and exclusion criteria to the incident JIA populations, we found a total of 121 polyJIA patients in the InGef database (65 diagnosed in 2014 and 56 in 2015) and 58 polyJIA patients in the WIG2 database (29 diagnosed in 2014 and 29 in 2015).

### 3.1. Treatment Patterns: Most Commonly Prescribed (Top 20) and Predefined Drugs

During the baseline, the most frequently prescribed JIA-relevant drugs were NSAIDs such as ibuprofen, prescribed to 61.98% and 65.52% of patients in the InGef and WIG2 datasets, respectively. The second most often prescribed were pain medications (17.36% and 25.86%, respectively), followed by corticosteroids (13.22% and 13.79% of the InGef and WIG2 populations, respectively).

**Figure 2:**
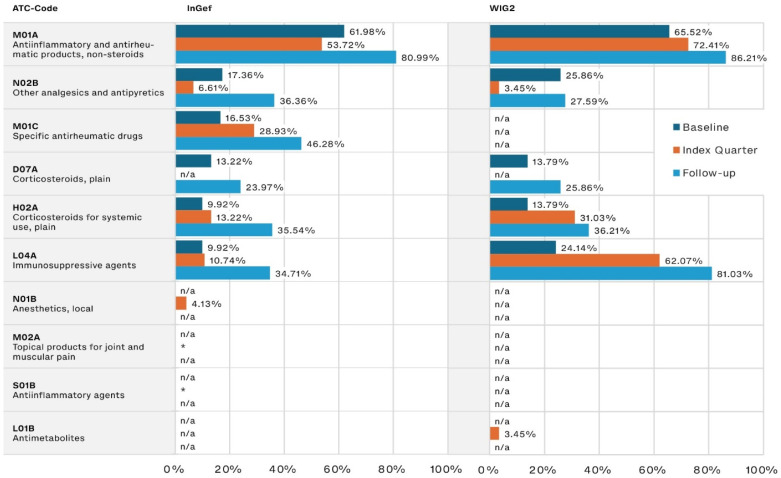
Disease-relevant drugs of the most frequently prescribed drugs, by timeframe relative to incident diagnosis, as pooled results (patients diagnosed in both 2014 and 2015) for InGef and WIG2 databases (in % of patients with a prescription); * indicates there were <5 patients, and therefore, no % could be calculated (note that because the top prescribed medications were analyzed for each of the three time periods, some medications were not one of the top medications prescribed in each timeframe and are marked with n/a).

NSAID use increased steadily into the follow-up period (80.99% and 86.21% in the InGef and WIG2 databases, respectively). DMARD use (referred to as specific antirheumatic drugs and immunosuppressive agents, including csDMARDs like methotrexate and nearly all bDMARDs), also increased from baseline to index quarter and then again into follow-up (Figure 2). Pain medication (referred to as other analgesics and antipyretics), and systemic corticosteroid use increased from baseline to follow-up, but the increase was less substantial than that of csDMARDs and bDMARDs (approximately 20 percentage points in each dataset, from baseline to follow-up).

**Figure 3:**
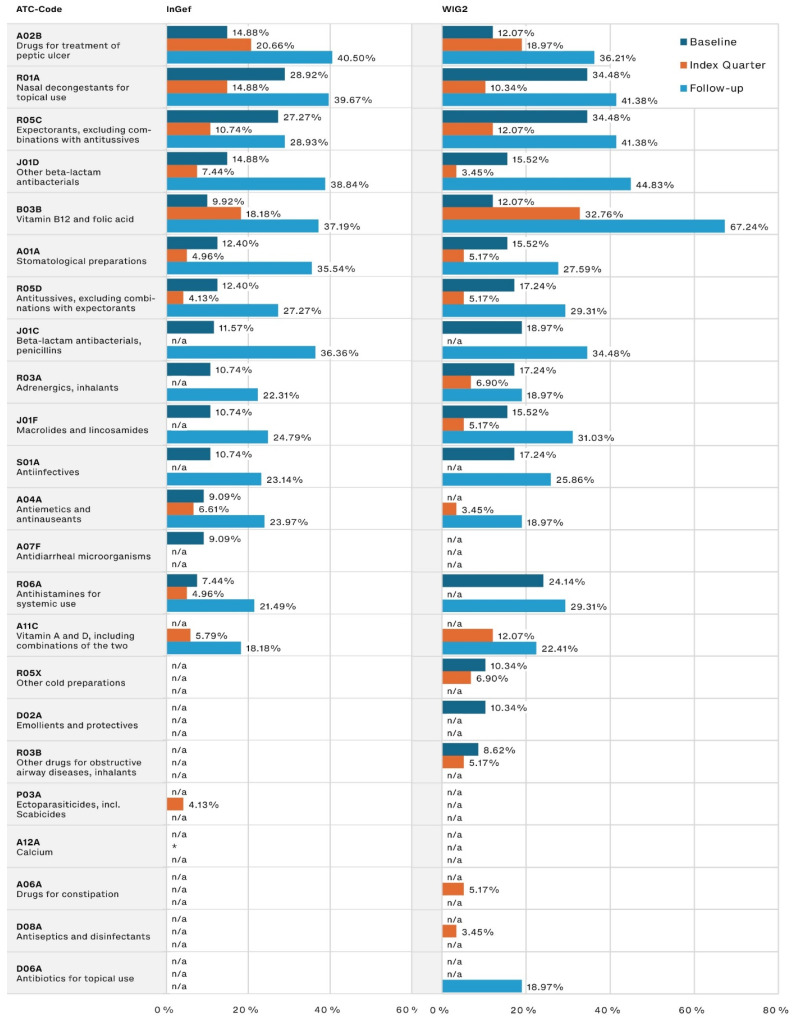
Other most frequently prescribed drugs, by timeframe relative to incident diagnosis, as pooled results (patients diagnosed in both 2014 and 2015) for InGef and WIG2 databases (in % of patients with a prescription); * indicates there were <5 patients in the sample, and therefore, no % could be calculated (note that because the top prescribed medications were analyzed for each of the three time periods, some medications on were not one of the top medications prescribed in each timeframe and are marked with n/a).

The most frequently prescribed other medications (Figure 3) were those used for peptic ulcer treatment, for which the prescription frequency in our population tripled from the baseline to the follow-up period (14.88% to 40.50% and 12.07% to 36.21% in the InGef and WIG2 datasets, respectively). Medications associated with infections (including nasal decongestants and expectorants) were among the most frequently prescribed during the baseline and follow-up, and the use of both penicillin and other beta-lactam antibacterials increased from baseline to follow-up, nearly tripling in prescription frequency.

We specifically analyzed the proportion of patients that were prescribed a medication in each predefined drug group; NSAIDs, GCs, csDMARDs, bDMARDs, and JAKis. During the baseline period, between 60.71% and 68.97% of patients were prescribed an NSAID, while between 6.90% and 20.69% were prescribed a GC. During the baseline, more patients were prescribed a csDMARD (between 13.79% and 27.59%) than a GC, with a substantial increase in this difference during the index quarter and into follow-up, where a csDMARD was prescribed to nearly twice as many patients as were prescribed a GC (see Supplemental Table S6).

In each database, we see the proportion of patients being prescribed a csDMARD or a bDMARD during index and follow-up from 2014 to 2015 increase. There was an increase in csDMARD prescriptions from 55.17% in 2014 to 68.97% in 2015 during the index quarter (WIG2) and an increase from 46.15% in 2014 to 51.79% in 2015 in the follow-up (InGef), with increases of similar magnitude in bDMARD prescriptions in WIG2 data. bDMARD prescriptions, however, decreased among InGef database patients from 2014 to 2015 (10.77% to 10.71% in the index and 33.85% to 25.00% in follow-up).

There were no JAKi prescriptions in either database, in any of the observed timeframes; therefore, we will no longer refer to JAKi data throughout the paper.

We found up to four different csDMARDs were prescribed in the WIG2 dataset, despite most patients in this dataset having been prescribed methotrexate, and up to four different bDMARDs (both datasets) (see Supplemental Table S7).

### 3.2. Treatment Patterns: Time to Prescriptions

It took an average of 128 days (114 and 141 in WIG2 and InGef databases, respectively), from the time of the first polyJIA diagnosis to the first csDMARD prescription (95% confidence interval (CI): 57.7–171.1, range 1–1325 days in the WIG2 dataset; 95% CI: 92.2–190.7, range 1–906 in the InGef dataset) and an average of 327 days (361 and 292 days in WIG2 and InGef databases, respectively) from the first polyJIA diagnosis to a first bDMARD prescription (95% CI: 237.7–484.3, range 34–1183 days in the WIG2 dataset; 95% CI: 188.6–395.9, range 0–1178 days in the InGef dataset). There was an average of 243 days (288 and 198 days in WIG2 and InGef databases, respectively) between a first csDMARD and a first bDMARD prescription (95% CI: 170.2–406.3 in the WIG2 dataset; 95% CI: 69.5–326.6 in the InGef dataset).

**Figure 4:**
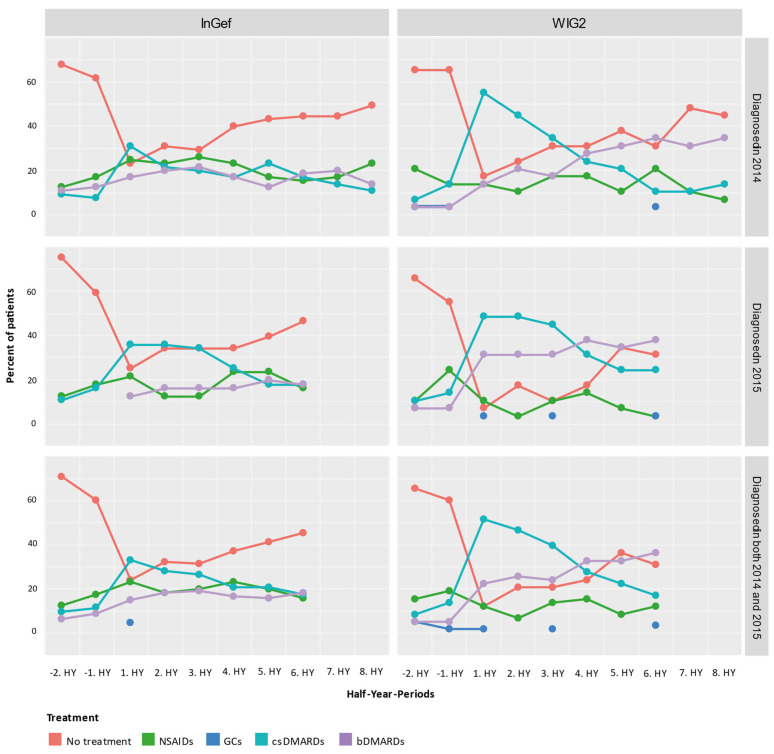
Cross-sectional analysis of the drug treatment escalation levels over time (HY = half-year); NSAIDs: non-steroidal anti-inflammatory drugs; GCs: glucocorticoids; csDMARDs: conventional synthetic disease-modifying antirheumatic drugs; bDMARDs: biological disease-modifying antirheumatic drugs.

We rated the treatment of pooled patients diagnosed in 2014 and 2015 and analyzed them cross-sectionally in HY intervals from their individual index quarters in both baseline and follow-up directions (Figure 4). At one year before the first diagnosis (i.e., index quarter), most patients were not treated with any of the predefined medications (71.07% in InGef dataset and 65.52% in WIG2), but of those who were, most were treated with NSAIDs (12.40% in InGef dataset and 15.52% in WIG2). We also see some patients already had received a prescription for csDMARDs and bDMARDs before being diagnosed with polyJIA. Although absolute numbers were low (<5 patients in the WIG2 dataset and 12 and 8 patients in InGef data receiving csDMARD and bDMARD, respectively), this still amounted to 10% and 6% in the InGef dataset.

In the first HY period after the index, most patients in the population were treated with a csDMARD without a bDMARD. There were still some patients (23.97% and 12.07% of pooled patients in InGef and WIG2 data, respectively) receiving none of the investigated treatments at this time. After about 3 years of follow-up, the largest group of patients were not being prescribed any of the treatments (45.45% and 31.03%); however, some (18.18% and 36.21%) were prescribed bDMARDs.

**Figure 5:**
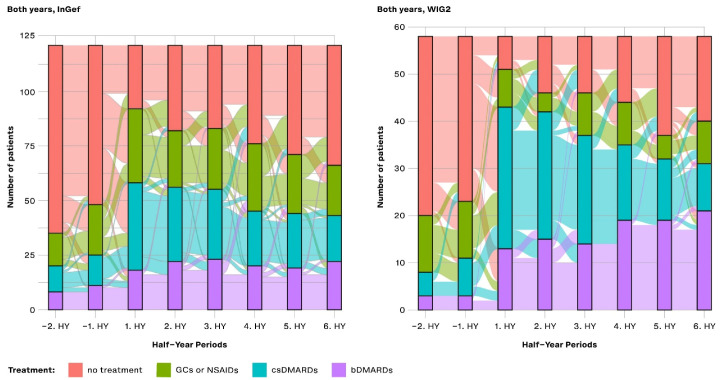
Longitudinal analysis from the InGef and WIG2 databases of drug treatment escalation at and throughout each time period for pooled patients diagnosed in 2014 and 2015; GCs and NSAIDs values are aggregated (HY = half-year).

Overall, the pattern of results of the longitudinal analysis (Figure 5) mirrors the pattern of results shown in the cross-sectional analysis: prior to diagnosis, most patients received no treatment, and then there was an increase in the proportion of patients treated with csDMARDs, followed by an increasing proportion of patients with either no treatment or treatment with a bDMARD. From −2HY during the baseline (a year prior to diagnosis) towards the index quarter, most patients with no treatment started a csDMARD, and few started a bDMARD. After the index quarter, however, some patients stopped their csDMARD, started taking a bDMARD, or stopped any of these treatments (no treatment), but most continued their csDMARD treatment.

Most patients that started bDMARDs at 1HY following the index continued to take them through to the 6HY period (three years post-index), with very few switching to csDMARDs or stopping treatment.

In both databases, there was an increase in the number of patients starting a csDMARD at 1HY following the index quarter, and around the same time, there was a noticeable increase in the use of bDMARDs, the use of which slightly increased through to the end of follow-up. There was a trend towards no treatment following the 1HY after the index. In the WIG2 database, this was primarily due to stopping csDMARD therapy until about 4HY post-index, when there were more patients stopping NSAID/GC. In the InGef database, it was mostly patients stopping NSAID/GC therapy who contributed to the increasing group of patients receiving no treatment, with less pronounced changes in csDMARDs as the highest escalation group.

Furthermore, most patients that started bDMARDs at 1HY following the index continued to take them through to the 6HY period (3 years post-index), with very few switching to the other options or stopping treatment. However, even before to after 1HY around the index, some csDMARD users had started taking a bDMARD.

### 3.3. Duration of Drug Exposure

**Figure 6:**
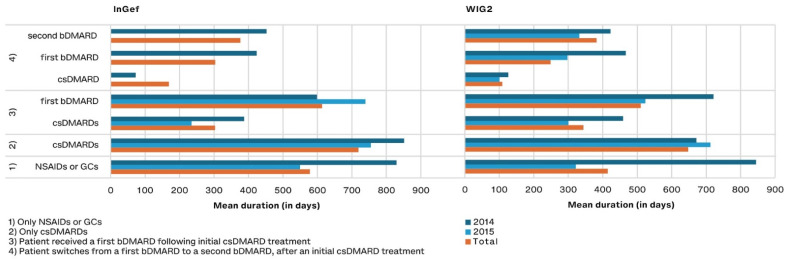
Drug exposure duration in days for predefined treatment patterns, by year of diagnosis (and pooled results) and database.

Of patients treated with DMARDs, those treated with csDMARDs as monotherapy remained on the drug the longest (a mean of 648 and 718 days with 95% CI: 511.0–784.5 and 603.3–833.3 in pooled WIG2 and InGef results, respectively) (Figure 6). Patients who were treated with a csDMARD and then a bDMARD took the csDMARD alone for about half as long as they took the bDMARD (303 and 614 days in the InGef with 95% CI: 164.6–441.2 and 460.3–766.7; 344 and 510 days in the WIG2 dataset with 95% CI: 197.2–491.4 and 361.2–659.1). When a second bDMARD was prescribed, the first bDMARD was taken for 303 and 248 days (95% CI: 73.7–533.1 and −84.1–580.6), and the second for 376 and 382 days (95% CI: 148.0–604.8 and −17.8–781.3 in InGef and WIG2 data, respectively) (see Supplemental Table S8).

## 4. Discussion

This is the first study presenting real-world healthcare claims data in a descriptive analysis of time to treatment from incident polyJIA diagnosis and changes in medications over time. This analysis used two large healthcare claims database samples to investigate treatment practices between 2014 and 2018 in incident polyJIA patients with RF+ or RF− polyarthritis or extended oligoarthritis diagnosis. Depending on the database and diagnosis year, between 58 and 121 patients were included in this analysis.

We found the real-world treatment patterns of these patients generally reflect guidelines [2]. While treatment with csDMARDs and/or bDMARDs was implemented in a timely manner in a substantial proportion of newly diagnosed polyJIA patients, a notable proportion were not receiving these treatments following diagnosis, and a large proportion were not being treated after three years of follow-up.

### 4.1. Top 20 Medications

The most frequently prescribed drugs were mostly JIA-specific (NSAIDs and DMARDs), with substantially increasing use from baseline to follow-up. While the substantial increase from baseline into the index quarter and follow-up is expected, it was surprising that up to 17% of patients were already receiving specific antirheumatic medications, even before their incident diagnosis. Since our incident polyJIA population was defined as not having met our polyJIA definition in the four quarters prior to index diagnosis, it was possible that these patients initially had other JIA diagnoses and symptoms prior to their incident polyJIA diagnosis and therefore were already being treated with antirheumatic drugs at the time of the incident polyJIA diagnosis. Other frequently prescribed medications were not directly related to JIA; however, they were related to medications used to treat JIA, such as vitamin supplements or peptic ulcer medications, perhaps needed with the use of methotrexate and NSAIDs [2,27,28]. The proportion of patients prescribed medications related to infections (especially antibiotics) also increased substantially during the follow-up period.

One important finding in our study is that the proportion of newly diagnosed polyJIA patients treated with csDMARDs and bDMARDs during the index quarter and follow-up increased from 2014 to 2015, suggesting a shift in JIA treatment even in our short 2-year incident patient observation period, which has also been observed in earlier studies [15]. The only exception was the proportion of patients receiving bDMARD prescriptions that decreased in the InGef dataset. This is particularly relevant, given evidence that earlier and more aggressive treatment of polyJIA may result in better outcomes [29]. What we did not observe, however, was a parallel decrease in other medications (NSAIDs, GCs, or csDMARDs) with the increase in bDMARD use, which has been shown in other studies [30], perhaps reflective of increasingly aggressive treatment in polyJIA patients.

### 4.2. Treatment Patterns

Due to the comparatively poor prognosis in polyJIA patients, German treatment guidelines recommend starting the csDMARD methotrexate immediately after diagnosis [2]. Studies have shown that early and aggressive treatment may improve outcomes [29,31]. We found that an average of 128 days went by between an initial polyJIA diagnosis and a first csDMARD prescription, and even longer to a first bDMARD prescription (from both a polyJIA diagnosis and a csDMARD prescription). Other studies have demonstrated much earlier use of DMARDs, with a median of 0.3 and 1.5 months in RF+ and RF− polyJIA patients, respectively [15]. Another large US American database study found that treatment initiation was 11 months shorter for non-biologic users, compared to biologic users who were also treated with a non-biologic [32].

In our cross-sectional analysis, we observed around 20% of patients not treated with any DMARD. While NSAIDs and a limited course of GCs can be used to manage symptoms during the initiation of DMARDs or biologics [33], these patients in our analysis had not been prescribed a DMARD at all. This indicates a gap between treatment guidelines and treatment in practice. Patients treated outside of specialized centers may not be treated as intensively and strictly as guidelines suggest [2].

We found a decrease in the proportion of patients with csDMARDs as the highest treatment escalation at 2HY following diagnosis in the InGef dataset, and the biggest proportion of patients were categorized as having no treatment. In the WIG2 database, this was the largest group at 5HY after the index. Nonetheless, the proportion of patients taking a bDMARD increased steadily, as the proportion of those for whom csDMARDs was the highest medication escalation decreased. Our cross-sectional analysis of the highest escalation of medications reached at each timepoint reflects results seen in other studies from different regions [15].

In our longitudinal analysis, we analyzed treatment switches or escalations over time. As in the cross-sectional analysis, we observed an increase in the proportion of patients taking csDMARDs at 1HY after diagnosis, as per polyJIA treatment recommendations [34], instead of an immediate bDMARD prescription. As follow-up continues, there is a slight trend towards escalating from csDMARD to bDMARD use. Few patients de-escalated to NSAIDs/GCs or to no treatment between 1HY and 2HY (WIG2) and 4HY after diagnosis (InGef). The increase in the proportion of patients taking a bDMARD seems to be due mostly to patients either stopping or adding onto their csDMARD therapy.

We did not distinguish between the ILAR categories in our polyJIA population and, therefore, we cannot determine what percentage of our polyJIA population corresponds to each JIA category. Other studies have observed that children with RF+ polyarthritis rarely discontinue treatment, suggesting that even early aggressive treatment may not result in remission [15]. Patients with polyJIA forms spend more time under treatment before achieving inactive disease than those with other JIA forms; one study reported there were 22, 29, and 23 months of active disease before the first episode of inactive disease in patients with extended oligoarthritis, RF+ polyarthritis, and RF− polyarthritis, respectively [35].

In our polyJIA patient population, we found that bDMARD use was highest during follow-up, with 25.0% and 51.7% of patients (InGef and WIG2 database, respectively) being prescribed a bDMARD. A study from Turkey observed rates from 41.5% to 64.7% in polyJIA patients diagnosed between 2010 and 2017, depending on the polyJIA ILAR category [36], further highlighting international differences.

We found that patients spent around 500–600 days on bDMARDs after previous treatment with a csDMARD. This is comparable to other studies, where the mean duration of biologic use was 17.3 ± 13.0 months in polyJIA patients [36]).

Since time spent with active disease early on in the course of JIA seems to be a significant factor in the future course of the disease, this long-term and aggressive treatment is important; a multi-national retrospective observational study found a significant association between time spent in active disease in the first two years and the future duration of active disease (years 3–5, *p* < 0.001) in 605 patients with oligoarthritis, polyarthritis, and systemic JIA in the Netherlands, Belgium, Germany, and Switzerland, diagnosed after 1991. The study found that time spent on active disease was the most significant factor associated with time spent with active disease in the following years; therefore, achieving inactive disease, even if remission is not attainable, is important [37].

### 4.3. Limitations

Our study results should be considered in light of limitations inherent to claims data studies. Since billing data are not collected for epidemiological studies, potential data entry or coding errors cannot be ruled out. There is no access to laboratory or clinical data such as rheumatoid factor, inflammation markers, or counts of tender and swollen joints, to confirm claims documentation. Finally, only patients who sought medical care or advice in the given timeframe were analyzed.

Since the definition of polyJIA can be different in other studies, including at least four or five joints [12], or including psoriatic arthritis, a comparison of our polyJIA results to other studies must be performed with caution. While our incident polyJIA population did not meet the polyJIA definition in the four quarters prior to polyJIA diagnosis, patients may have been previously (before these four quarters) diagnosed. This may have resulted in a partly prevalent polyJIA population. Furthermore, we selected patients based on diagnosis codes within the respective calendar year (1st of January to 31st of December), including patients with either a main inpatient diagnosis code or at least two verified outpatient codes in two different quarters in the year. Should a patient have been diagnosed twice (verified outpatient) in different quarters across calendar years (for example, in Q4 of 2014 and Q1 of 2015), we would not have captured this patient in our analysis, possibly underestimating polyJIA patients.

The research databases used for the analyses are representative of the German SHI population in terms of age and sex [23]; however, regional representation is not guaranteed. The two databases we used may have different regional representations, and region has been observed in other studies to play a role in JIA prevalence rates [38] and even JIA treatment habits [39]. Additionally, with low case numbers attributable to the rarity of the disease, the patient pool from which we draw any conclusions is limiting. The extent of missing data due to incomplete insurance, however, is very small, and the impact on the analyses is therefore negligible. Since health insurance billing data do not indicate socioeconomic status accurately regarding income, education, or occupation, this potential bias could not be considered in our analyses. Descriptive analyses based on health insurance data were performed, and statements on causal relationships are not feasible due to the small sample size for this rare childhood disease.

Billing data are not affected by health care providers’ or patients’ willingness to participate, as data are anonymous and consent for research purposes is not required. However, data protection laws require that categories for which the patient number is less than five may not be identified as such. This further limits our results in a population with this rare disease.

## 5. Conclusions

We found that many polyJIA patients started on csDMARDs relatively soon after diagnosis. However, despite the poor prognosis of polyJIA patients, a substantial proportion ended up on no treatment at the end of the three-year follow-up period. After three years of therapy, many patients still remain on csDMARD or bDMARD therapy, some even with a second bDMARD. This indicates sub-optimal response or intolerance of the treatments and a need for more treatment options. In our study, a substantial number of patients with polyJIA are not treated as intensively as guidelines recommend.

## Data Availability

The datasets generated and analyzed during the current study are not publicly available due to data protection laws. Raw dataset data are not publicly available to preserve individuals’ privacy under the European General Data Protection Regulation. No new data were created or analyzed in this study. Data sharing is not applicable to this article.

## Supplementary Materials

The following supporting information can be downloaded at: https://www.mdpi.com/article/10.3390/life14060712/s1: Table S1. ATC codes for NSAIDs used in the present study; Table S2. ATC codes for GCs used in the present study; Table S3. ATC codes for csDMARDs used in the present study; Table S4. ATC codes for bDMARDs used in the present study; Table S5. ATC codes for JAKi agents used in the present study; Table S6. Patients (% of total cohort) taking predefined medication group during each timeframe, by database, in 2014 and 2015; Table S7. Number of different prescribed DMARDs for each of cohorts 2014 and 2015 during the index period and the follow-up period based on the WIG2 database; Table S8. Drug exposure duration in days for each treatment group and therapy, split by cohort and database.
