# Supplementary material for: Treatment Patterns in Polyarticular Juvenile Idiopathic Arthritis: A Retrospective Observational Health Claims Data Study
Source: Life (Basel). 2024 May 31;14(6):712. doi: 10.3390/life14060712 (PMC11205221; doi:10.3390/life14060712)
Supplement: Supplementary file 1 [file life-14-00712-s001.zip › Supplemental Material [Table_S1].pdf]

Table S1. ATC codes for NSAIDs used in the present study

NSAIDs	*ATC code*
Naproxen	*M01AE02*
	M02AA12
	G02CC02
	M01AE52
Ibuprofen	*M01AE01*
	M02AA13
	C01EB16
	G02CC01
	R02AX02
	M01AE51
	N02AJ08
	N02AJ19
Indometacin	*M01AB01*
	M02AA23
	C01EB03
	S01BC01
	M01AB51
	M02AA73
Diclofenac	*M01AB05*
	M02AA15
	D11AX18
	S01BC03

	M01AB55
	N02AJ05
Etoricoxib	*M01AH05*
Celecoxib	*M01AH01*
	L01XX33
Piroxicam	*M01AC01*
	M02AA07
	S01BC06
Tenoxicam	*M01AC02*
Meloxicam	*M01AC06*
	M01AC56
Lornoxicam	*M01AC05*
Dexibuprofen	*M01AE14*
Flurbiprofen	*M01AE09*
	M02AA19
	R02AX01
	S01BC04
Ketoprofen	*M01AE03*
	M02AA10
	M01AE53
Tiaprofenic acid	*M01AE11*
