# Supplementary material for: Treatment Patterns in Polyarticular Juvenile Idiopathic Arthritis: A Retrospective Observational Health Claims Data Study
Source: Life (Basel). 2024 May 31;14(6):712. doi: 10.3390/life14060712 (PMC11205221; doi:10.3390/life14060712)
Supplement: Supplementary file 1 [file life-14-00712-s001.zip › Supplemental Material [Table_S2].pdf]

Table S2. ATC codes for GCs used in the present study

GCs	ATC code
Prednisone	H02AB07
Prednisolone	H02AB06
	H02BX06
	H02AB56
Methylprednisolone	H02AB04
	H02BX01
	H02AB54
Triamcinolone	H02AB08
	H02BX08
	H02AB58
Dexamethasone	H02AB02
	H02BX02
Betamethasone	H02AB01
	H02BX09
	H02AB51
Rimexolone	H02AB12
