# Supplementary material for: Treatment Patterns in Polyarticular Juvenile Idiopathic Arthritis: A Retrospective Observational Health Claims Data Study
Source: Life (Basel). 2024 May 31;14(6):712. doi: 10.3390/life14060712 (PMC11205221; doi:10.3390/life14060712)
Supplement: Supplementary file 1 [file life-14-00712-s001.zip › Supplemental Material [Table_S3].pdf]

**Table S3. ATC codes for csDMARDs used in the present study**

| csDMARDs | ATC code |
|---|---|
| Sulfasalazine | M01CX02 |
| | A07EC01 |
| Methotrexate | M01CX01 |
| | L01BA01 |
| | L04AX03 |
| Hydroxychloroquine | P01BA02 |
| Azathioprine | L04AX01 |
| Leflunomide | L04AA13 |
| Ciclosporine | L04AD01 |
| Cyclophosphamide | L01AA01 |
| Mycophenolic acid | L04AA06 |
| Gold preparations | M01CB* |
