# Supplementary material for: Treatment Patterns in Polyarticular Juvenile Idiopathic Arthritis: A Retrospective Observational Health Claims Data Study
Source: Life (Basel). 2024 May 31;14(6):712. doi: 10.3390/life14060712 (PMC11205221; doi:10.3390/life14060712)
Supplement: Supplementary file 1 [file life-14-00712-s001.zip › Supplemental Material [Table_S4].pdf]

Table S4. ATC codes for bDMARDs used in the present study

bDMARDs	ATC code
Etanercept	L04AB01
Adalimumab	L04AB04
Anakinra	L04AC03
Canakinumab	L04AC08
Tocilizumab	L04AC07
Abatacept	L04AA24
Golimumab	L04AB06
Infliximab	L04AB02
Rituximab	L01XC02
