# Supplementary material for: Treatment Patterns in Polyarticular Juvenile Idiopathic Arthritis: A Retrospective Observational Health Claims Data Study
Source: Life (Basel). 2024 May 31;14(6):712. doi: 10.3390/life14060712 (PMC11205221; doi:10.3390/life14060712)
Supplement: Supplementary file 1 [file life-14-00712-s001.zip › Supplemental Material [Table_S5].pdf]

Table S5. ATC codes for JAKi agents used in the present study

JAKi	ATC code
**Tofacitinib**	L04AA29
**Baricitinib**	L04AA37
