# Supplementary material for: Treatment Patterns in Polyarticular Juvenile Idiopathic Arthritis: A Retrospective Observational Health Claims Data Study
Source: Life (Basel). 2024 May 31;14(6):712. doi: 10.3390/life14060712 (PMC11205221; doi:10.3390/life14060712)
Supplement: Supplementary file 1 [file life-14-00712-s001.zip › Supplemental Material [Table_S6].pdf]

Table S6. Patients (% of total cohort) taking pre-defined medication group during each timeframe, by database, in 2014 and 2015

Drug group	Timeframe	Baseline, rate (%)		Index Q, rate (%)		Follow-up, rate (%)	
	**Database**	**2014**	**2015**	**2014**	**2015**	**2014**	**2015**
**NSAIDs**	InGef	64.62%	60.71%	52.31%	57.14%	89.23%	71.43%
	WIG2	65.52%	68.97%	72.41%	72.41%	82.76%	93.10%
**GCs**	InGef	7.69%	12.50%	10.77%	16.07%	38.46%	30.36%
	WIG2	6.90%	20.69%	20.69%	41.38%	34.48%	41.38%
**csDMARDs**	InGef	18.46%	21.42%	27.69%	33.92%	46.15%	51.79%
	WIG2	13.79%	27.59%	55.17%	68.97%	75.86%	86.21%
**bDMARDs**	InGef	12.31%	<5	10.77%	10.71%	33.85%	25.00%
	WIG2	3.45%	6.90%	10.34%	17.24%	44.83%	51.72%
