# Supplementary material for: Treatment Patterns in Polyarticular Juvenile Idiopathic Arthritis: A Retrospective Observational Health Claims Data Study
Source: Life (Basel). 2024 May 31;14(6):712. doi: 10.3390/life14060712 (PMC11205221; doi:10.3390/life14060712)
Supplement: Supplementary file 1 [file life-14-00712-s001.zip › Supplemental Material [Table_S7].pdf]

Table S7. Number of different prescribed DMARDs for each of cohorts 2014 and 2015 during the index period and the follow-up period based on the WIG2 database.

Note that for Cohort 2014 this time period consists of 17 quarters whereas for Cohort 2015, this time period consists of 13 quarters. Values indicate absolute counts, percentages are shown in parentheses (N = number of DMARDs, SD = standard deviation)

	Cohort 2014				Cohort 2015			
	N csDMARDs		N bDMARDs		N csDMARDs		N bDMARDs	
	InGef	WIG2	InGef	WIG2	InGef	WIG2	InGef	WIG2
**Mean**	1.36	1	1.5	0.55	1.41	1.03	1.14	0.79
**Median**	1	1	1	0	1	1	1	1
**SD**	0.55	0.80	0.91	0.69	0.57	0.50	0.36	1.05
**Min**	1	0	1	0	1	0	1	0
**Max**	3	4	4	2	3	2	2	4
**0.25 quantile**	1	1	1	0	1	1	1	0
**0.75 quantile**	2	1	1.75	1	2	1	1	1
