# Supplementary material for: Treatment Patterns in Polyarticular Juvenile Idiopathic Arthritis: A Retrospective Observational Health Claims Data Study
Source: Life (Basel). 2024 May 31;14(6):712. doi: 10.3390/life14060712 (PMC11205221; doi:10.3390/life14060712)
Supplement: Supplementary file 1 [file life-14-00712-s001.zip › Supplemental Material [Table_S8].pdf]

Table S8. Drug exposure duration in days for each treatment group and therapy, split by cohort and database

Year	Treatment Group	Therapy	n patients InGef	Mean days InGef	n patients WIG2	Mean days WIG2
**2014**	Only NSAIDs or GCs	NSAIDs or GCs	21	829	2	844
	Only csDMARDs	csDMARDs	13	851	10	671
	Patient received a first bDMARD following initial csDMARD treatment	first bDMARD	14	599	9	721
		csDMARDs	9	387	9	458
	Patient switches from a first bDMARD to a second bDMARD, after an initial csDMARD treatment	first bDMARD	8	423	2	467
		second bDMARD	6	452	2	422
		csDMARD	5	73	2	126
**2015**	Only NSAIDs or GCs	NSAIDs or GCs	20	549	3	322
	Only csDMARDs	csDMARDs	17	755	11	712
	Patient received a first bDMARD following initial csDMARD treatment	first bDMARD	11	739	10	523
		csDMARDs	7	235	9	300
	Patient switches from a first bDMARD to a second bDMARD, after an initial csDMARD treatment	first bDMARD	<5	-	3	297
		second bDMARD	<5	-	3	332
		csDMARD	<5	-	4	101
**Total**	Only NSAIDs or GCs	NSAIDs or GCs	40	578	6	415
	Only csDMARDs	csDMARDs	31	718	20	648
	Patient received a first bDMARD following initial csDMARD treatment	first bDMARD	25	614	18	510
		csDMARDs	15	303	17	344

	Patient switches from a first bDMARD to a second bDMARD, after an initial csDMARD treatment	first bDMARD	9	303	4	248
		second bDMARD	7	376	4	382
		csDMARD	7	169	6	109
